# IT’S THE LITTLE THINGS THAT COUNT: IMPLICATIONS OF DAILY EXPERIENCES FOR WELL-BEING AND BIOLOGICAL INDICATORS

**DOI:** 10.1093/geroni/igz038.2712

**Published:** 2019-11-08

**Authors:** Kira S Birditt

**Affiliations:** Institute for Social Research, University of Michigan, Ann Arbor, Michigan, United States

## Abstract

Middle-age and older adults vary widely in their physical health. This symposium describes studies that identify diverse daily experiences that account variation in health using multiple indicators of well-being (self-reported, biological). Fingerman et al. assessed daily TV viewing among older adults and found that more frequent television watching was associated with poorer physical health, worse health behaviors, and less energy expenditure (via actical watch). Leger et al., examined links between daily affect and sleep. Greater variability in daily positive affect is associated with fewer hours of sleep and greater morning tiredness even after adjusting for mean levels of affect. Luong et al. examined links between daily stress and affect. Interpersonal stressors were associated with greater affect reactivity than non-interpersonal stressors and links were reduced among older adults. Birditt et al. assessed links between daily social interactions and cardiovascular reactivity. More frequent social interactions and negative social interactions were associated with increased heart rate and links varied by gender and race. Polenick et al. examined links between daily social interactions and salivary DHEA-S (a marker of the stress response). Positive interactions predicted greater DHEA-S over the course of the day and links between negative interactions and DHEA-S varied by age group such that younger individuals appeared to be more reactive. These studies offer important clues regarding how daily experiences get under the skin to influence health and well-being.

